# Generation of Transmission-Competent Human Malaria Parasites with Chromosomally-Integrated Fluorescent Reporters

**DOI:** 10.1038/s41598-019-49348-x

**Published:** 2019-09-11

**Authors:** Kyle Jarrod McLean, Judith Straimer, Christine S. Hopp, Joel Vega-Rodriguez, Jennifer L. Small-Saunders, Sachie Kanatani, Abhai Tripathi, Godfree Mlambo, Peter C. Dumoulin, Chantal T. Harris, Xinran Tong, Melanie J. Shears, Johan Ankarklev, Björn F. C. Kafsack, David A. Fidock, Photini Sinnis

**Affiliations:** 1 Department of Molecular Microbiology and Immunology, and Johns Hopkins Malaria Institute, Johns Hopkins Bloomberg School of Public Health, Baltimore, MD 21205 USA; 2 Department of Microbiology and Immunology, Columbia University Irving Medical Center, New York, NY 10032 USA; 3 Division of Infectious Diseases, Department of Medicine, Columbia University Irving Medical Center, New York, NY 10032 USA; 4 Department of Microbiology and Immunology, Weill Cornell School of Medicine, New York, NY 10065 USA; 5 Present Address: Department of Biological Engineering, Massachusetts Institute of Technology, Cambridge, MA 02139 USA; 6 Present Address: Novartis Institutes for Biomedical Research, Novartis Institute for Tropical Diseases, Emeryville, CA 94608 USA; 7 Present Address: Laboratory of Immunogenetics, National Institute of Allergy and Infectious Diseases, National Institutes of Health, Rockville, Maryland USA; 8 Present Address: Laboratory of Malaria and Vector Research, National Institute of Allergy and Infectious Diseases, National Institutes of Health, Rockville, MD 20892 USA; 9 Present Address: Department of Immunology and Infectious Diseases, Harvard T.H. Chan School of Public Health, Boston, Massachusetts 02115 USA; 10 Present Address: Center for Emerging and Re-emerging Infectious Diseases, University of Washington, Seattle, WA 98109 USA; 11 Present Address: Department of Molecular Biosciences, The Wenner Gren Institute, Stockholm University, SE-106 91 Stockholm, Sweden

**Keywords:** Drug screening, Parasitology, Vaccines

## Abstract

Malaria parasites have a complex life cycle that includes specialized stages for transmission between their mosquito and human hosts. These stages are an understudied part of the lifecycle yet targeting them is an essential component of the effort to shrink the malaria map. The human parasite *Plasmodium falciparum* is responsible for the majority of deaths due to malaria. Our goal was to generate transgenic *P*. *falciparum* lines that could complete the lifecycle and produce fluorescent transmission stages for more in-depth and high-throughput studies. Using zinc-finger nuclease technology to engineer an integration site, we generated three transgenic *P*. *falciparum* lines in which *tdtomato* or *gfp* were stably integrated into the genome. Expression was driven by either stage-specific *peg4* and *csp* promoters or the constitutive *ef1a* promoter. Phenotypic characterization of these lines demonstrates that they complete the life cycle with high infection rates and give rise to fluorescent mosquito stages. The transmission stages are sufficiently bright for intra-vital imaging, flow cytometry and scalable screening of chemical inhibitors and inhibitory antibodies.

## Introduction

Malaria remains one of the most important infectious diseases in the world, impacting approximately a third of the world’s population and responsible for over 400,000 deaths annually^1^. The disease is caused by parasites of the genus *Plasmodium*, transmitted to their mammalian host by the infected Anopheline mosquitoes. Malaria parasites have two distinct stages in their mammalian hosts: an asymptomatic pre-erythrocytic stage, consisting of sporozoites and the liver stages into which they develop, and an erythrocytic stage when high parasite numbers lead to clinical disease. A small proportion of blood stage parasites differentiate to gametocytes which, when taken up in the blood meal, initiate infection in the mosquito, giving rise to oocysts on the midgut wall that produce sporozoites that migrate to the mosquito’s salivary glands. The transmission stages of *Plasmodium*, namely sporozoites and gametocytes, are bottlenecks in the parasite life cycle and their low numbers has limited investigations into their biology and chemotherapeutic interventions.

Rodent malaria models have enabled more in-depth study of the difficult to access transmission stages and have played a particularly important role in our understanding of pre-erythrocytic stages. Nonetheless, there are species-specific differences across the *Plasmodium* genus, such as the unique morphology and prolonged development of gametocytes of the *Laverania*, that make it critical to perform confirmatory studies and inhibitor screening with human malaria parasites. In order to facilitate these investigations, we generated transgenic *Plasmodium falciparum* lines expressing tdTomato or Green Fluorescent Protein (GFP) under the control of pre-erythrocytic or gametocyte stage promoters. Here we describe the generation of these parasites and their fluorescent properties throughout the development of the transmission stages.

## Results and Discussion

### Generation of fluorescent *P*. *falciparum* lines

Previous studies have generated fluorescent *P*. *falciparum* lines using the constitutively expressed elongation factor *eEF1α* (PF3D7_1357000) promoter and GFP variants^2,3^. While useful, the low intensity of fluorescence of these parasites during sporozoite and exoerythrocytic stages has limited their utility, particularly when imaging in mosquito and mammalian tissues where there is a high degree of autofluorescence in the green channel. We reasoned that using a red fluorescent protein in the place of GFP could surmount some of these challenges, as the red-shifted fluorescence is better suited for intravital tissue imaging. We chose tandem-dimer Tomato (tdTomato)^4^ as its intrinsic brightness is nearly three times higher than that of GFP and six times higher than the commonly-used red fluorescent protein mCherry^5^.

Additionally, we reasoned that strong, stage-specific promoters could be used to further boost the fluorescent signal during the sporozoite and exoerythrocytic stage. We tested two potential candidates, the promoter of the circumsporozoite protein (CSP) gene (PF3D7_0304600), which drives expression of the most abundant sporozoite surface protein, and the promoter of the Plasmodium Early Gametocyte 4 (PEG4, also known as eTRAMP10.3) (PF3D7_1016900) gene. PEG4 was first described as a protein expressed in the early stages of gametocytogenesis^6^, and its promoter region has been shown to be active at those times^7^. Indeed, the utility of this promoter to drive episomal transgene expression in *P*. *falciparum* gametocytes has been previously demonstrated^7^. However, fluorescence in mosquito stages was never assessed as these studies used a line that does not make infection-competent gametocytes. More recently, PEG4 was reported to be the homolog of the well-studied *P*. *yoelii upregulated in sporozoites 4* (UIS4) gene^8^. The *uis4* promoter has been a valuable tool for driving strong fluorescence in sporozoites of *P*. *yoelii and P*. *berghei*^9^. We hypothesized that the same would be true for *P*. *falciparum*, potentially providing a promoter that could drive expression in both sporozoites and gametocytes.

**Figure 1:**
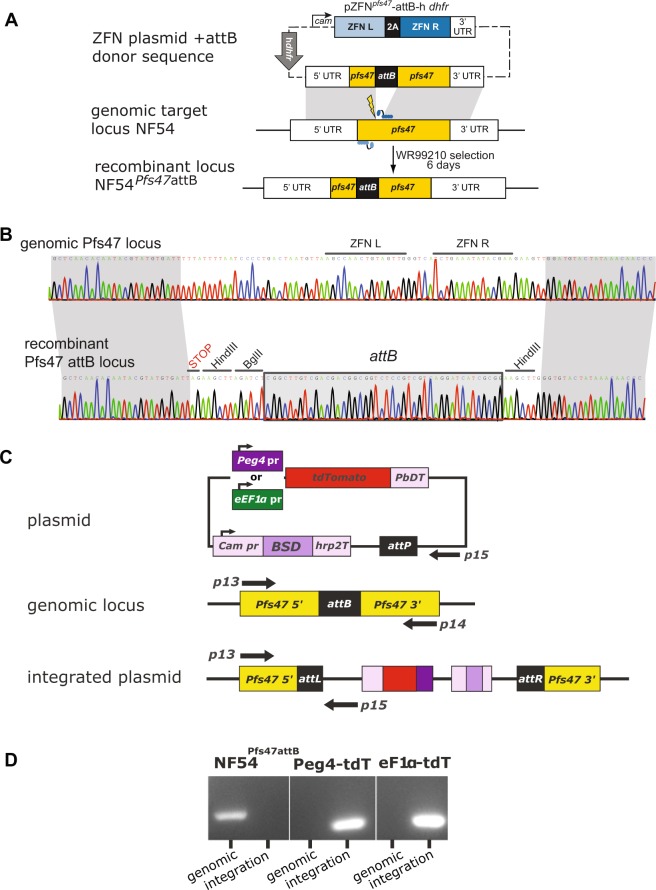
Introduction of the *attB* site into *pfs47* locus and integration of tdTomato transgenes. (**A**) Schematic of the *attB* integration strategy. The expression of the *pfs47*-specific ZFN pair is under the control of the *calmodulin* promoter and the *hsp86* 3′UTR. The homologous donor sequence provided for double-strand break repair comprises a fragment of *pfs47* stretching 0.68 kb upstream and 0.87 kb downstream of the ZFN target site (thunderbolt). The attB site is located between the two homologous regions and replaces the genomic *pfs47* sequence containing the ZFNs binding site upon integration. The transfection plasmid pZFN^*pfs47*^-attB-*hdhfr* also contains a selectable marker for transient selection and propagation of the plasmid. (**B**) Sequence analysis of the unmodified endogenous *pfs47* locus of the parental parasite line NF54 and the gene-edited recombinant locus NF54^pf47-attB^. (**C**) Schematic of the *tdTomato* plasmids used for transfection (*PbDT* = *P*. *berghei* DHFR-TS terminator, *Cam pr* = *calmodulin* promoter, *BSD* = Blasticidin S Deaminase selectable marker, *hrp2T* = HRP2 terminator) and of the NF54^Pfs47attB^ *attB* locus pre- and post-integration of the *tdTomato* plasmids. Position of primers used to amplify the genomic locus (p13 & p14) and the integrated plasmid (p13 & p15) are shown. (**D**) PCR verification of plasmid integration. Primers p13 and p14 generate a 334 bp product from the unmodified *pfs47attB locus* (genomic), and primers p13 and p15 generate at 287 bp product upon *tdTomato* plasmid integration (integration). Shown is one gel with lines between lanes added to delineate the different *Plasmodium* clones. Primer sequences in Table S1.

Typically, the production of genetically-modified *P*. *falciparum* parasites requires long *in vitro* selection and cloning times, which frequently results in the loss of the parasite’s ability to generate gametocytes or infect the mosquito. Beginning with a highly mosquito-infectious clone of the *P*. *falciparum* NF54 isolate^10^, we used zinc-finger nuclease (ZFN) technology to rapidly engineer a selectable marker-free *Bxb1* phage integration site (*attB*) into the parasite genome (Fig. 1A). We selected the coding sequence of the *pfs47* gene for *attB* introduction. Though recent work showed that Pfs47 is required for parasite evasion of the mosquito complement system in *Anopheles gambiae* mosquitoes^11^, it is not required for parasite survival in *Anopheles stephensi*^12,13^ and has been used by others as a locus for transgene expression in mosquito stages^2,3^. Once a stably-transfected parasite population was obtained, the line was cloned by limiting dilution, and the *pfs47* locus was sequenced to verify the presence of the *attB* site (Fig. 1B). Clones were subsequently tested for gametocyte production and mosquito infectivity.

A clone with high production of infectious gametocytes (NF54^Pfs47attB^ C10) was then selected for secondary transfection. Using the *Bxb1* integrase system^14^, a plasmid containing either Pf-*ef1α*-tdTomato, Pf-*peg4*-tdTomato, (Fig. 1C) or Pf-*csp*-GFP expression cassettes along with a Blasticidin S Deaminase (BSD) resistance cassette was introduced into the chromosomal *pfs47 attB* site. Stably transfected cultures were cloned by limiting dilution and tested for plasmid integration (Fig. 1D) and gametocyte production. Three clones of each of the tdTomato lines were passaged through mosquitoes to identify a clone producing robust sporozoite numbers. Pf-*peg4*-tdTomato Clone A and Pf-*ef1α*-tdTomato Clone 3D06 were chosen for further characterization. The Pf-*csp*-GFP line was not cloned and the uncloned line was studied.

Since these lines are the product of two sequential transfections and a limiting dilution cloning procedure, the tdTomato clones were further analyzed to determine whether they harbored the selectable marker cassettes used in each transfection. As expected, diagnostic PCRs performed with genomic DNA from both tdTomato lines had the BSD cassette (Supplementary Fig. 1). Because the *Bxb1 attB* site was introduced through marker-free homology directed repair, the episomal plasmid carrying the ZFNs and hDHFR marker was expected to have been lost after maintenance of the NF54^Pfs47attB^ C10 line in the absence of WR99210 pressure. Unexpectedly, PCRs revealed that the Pf-*ef1α*-tdTomato line retained both the hDHFR cassette and the zinc finger components, whereas the Pf-*peg4*-tdTomato line did not (Supplementary Fig. 1). We attribute this to the initial NF54^Pfs47attB^ C10 culture being comprised of two populations, one that had undergone ZFN-mediated recombination that integrated the *attB* locus without the remainder of the plasmid, and the other that underwent single-site crossover integration with the entire ZFN plasmid being integrated. The Pf-*peg4*-tdTomato clones were found by PCR to have been generated from parasites that had already lost the plasmid carrying the hDHFR cassette and the ZFNs, and thus carry only the BSD cassette. In contrast, the Pf-*ef1α*-tdTomato clones integrated into the parasites carrying the original ZFN plasmid and thus contain both the hDHFR and BSD cassettes (Supplementary Fig. 1).

To phenotypically confirm retention of the hDHFR selectable marker in the Pf-*ef1α*-tdTomato clone, we cultured blood-stage parasites of both tdTomato lines in the presence of 2.5 nM of the anti-folate antimalarial agent WR99210, which inhibits *P*. *falciparum* DHFR but allows hDHFR-expressing parasites to proliferate. In the absence of drug both lines grew well, were split on day 6, and continued their normal growth. In the presence of WR99210, the *peg4*-tdTomato clone began to die within the first 24 hr of drug pressure and no parasites were microscopically visible by day 3. We continued to follow the culture for an additional 9 days during which time no viable parasites were observed. In contrast, the Pf-*ef1α*-tdTomato parasites proliferated in the presence of 2.5 nM WR99210. While the retention of the hDHFR resistance cassette in Pf-*ef1α*-tdTomato clone will preclude its use for the discovery of novel anti-folate compounds, integration of the ZFN-expression plasmid should not interfere with other intended uses of this cell line.

### Phenotypic characterization of *P*. *falciparum* fluorescent lines

**Figure 2:**
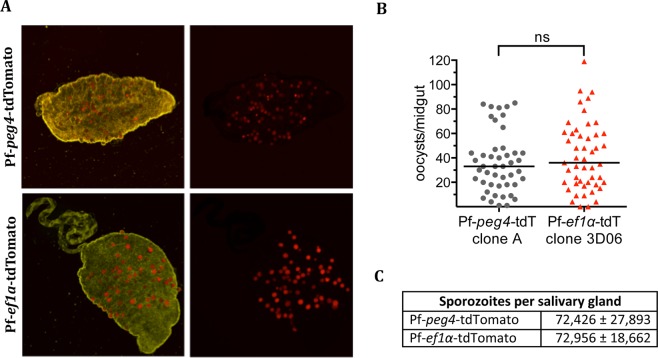
Passage through the mosquito of *P*. *falciparum*-tdTomato lines is normal. (**A**) Mosquito midguts (whitelight) were dissected 10 days after the infectious blood meal and imaged using fluorescence microscopy to visualize associated Pf-*peg4*-tdTomato and Pf-*ef1α*-tdTomato oocysts (red). (**B**) Oocysts were counted based on their tdTomato fluorescence and data from two independent feeding experiments each are shown. Horizontal line shows median oocyst number. Groups were compared using an unpaired t-test with equal standard deviations. (**C**) Pf-*peg4*-tdTomato and Pf-*ef1α*-tdTomato lines produce normal number of salivary gland sporozoites. Shown is the mean +/− standard deviation of salivary gland loads from 20 mosquitoes.

To characterize the tdTomato clones and the uncloned GFP line in the mosquito host, *Anopheles stephensi* mosquitoes were fed with human blood containing gametocytes and subsequent parasite development in mosquitoes was followed. Oocyst numbers for the tdTomato lines were robust and as expected, both Pf-*ef1α*-tdTomato and Pf-*peg4*-tdTomato oocysts were fluorescent (Fig. 2A,B). Indeed at this stage, Pf-*ef1α*-tdTomato fluorescence is quite strong, likely because each oocyst consists of many genomes. Oocyst numbers in the GFP line were somewhat lower than what we observed in the tdTomato lines, though this level of infectivity is still within the range that can be useful for experimental analyses (Supplementary Fig. 2A,B).

**Figure 3:**
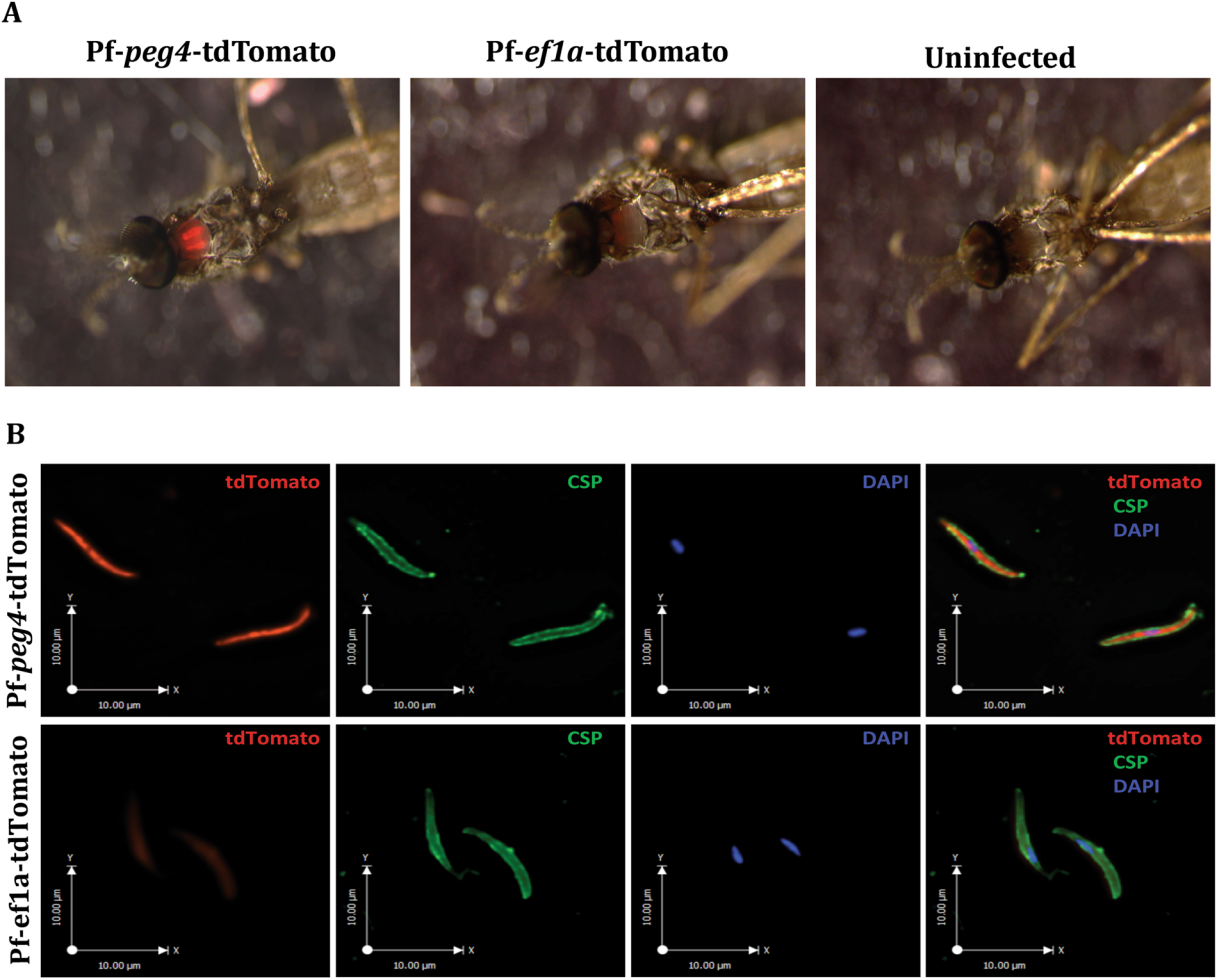
Immunofluorescence of sporozoites of *P*. *falciparum*-tdTomato lines. (**A**) The thorax of mosquitoes infected with the Pf-*ef1α*-tdTomato or Pf-*peg4*-tdTomato lines was visualized under a fluorescence stereoscope to detect the presence of the tdTomato sporozoites in the salivary glands. Note the red fluorescence observed in the thorax of mosquitoes infected with the *P*. *falciparum*-tdTomato lines compared to the absence of fluoresce in the uninfected mosquito. (**B**) Salivary gland sporozoites from mosquitoes infected with the Pf-*ef1α*-tdTomato or Pf-*peg4*-tdTomato were fixed and stained with an anti-PfCSP antibody (green) and analyzed by fluorescence microscopy to detect the cytoplasmic tdTomato (red) and the surface CSP (green). DNA was stained with DAPI (blue). Note the more intense signal detected from Pf-*peg4*-tdTomato sporozoites as compared to Pf-*ef1α*-tdTomato sporozoites, which match the fluorescence intensities from panel A.

In all three lines, oocyst sporozoites progressed to invade salivary glands with sporozoite numbers similar to those routinely observed in the NF54 parental line (Fig. 2C and Supplementary Fig. 2C). With both tdTomato lines we performed additional mosquito cycles, some in parallel with the NF54 parental line. The infectivity of these parasites for mosquitoes is consistent with that shown in Fig. 2: ef1α-tdTomato yielded a mean ± SEM of 48,934 ± 26,948 sporozoites per mosquito n = 3 cycles; peg4-tdTomato yielded 51,336 ± 6,581 sporozoites per mosquito n = 14 cycles; NF54 wild type yielded 33,116 ± 4,527 sporozoites per mosquito n = 6 cycles. As expected, salivary glands infected with Pf-*ef1α*-tdTomato sporozoites were dimly fluorescent, while Pf-*peg4*-tdTomato infected salivary glands were brightly fluorescent and could easily be visualized through the chitin exoskeleton of the mosquito (Fig. 3A), confirming that the *peg4* promoter is highly active in salivary gland sporozoites. We then performed immunofluorescence assays on salivary gland sporozoites to compare the endogenous tdTomato or GFP fluorescence with the signal observed upon immunostaining of the major surface protein, the circumsporozoite protein (CSP). The tdTomato signal when under the control of the *peg4* promoter gave the strongest fluorescent signal in sporozoites (Fig. 3B). GFP fluorescence under the *csp* promoter was robust but not as strong as that observed in the Pf-*peg*4-tdTomato sporozoites (Compare Fig. 3B and Supplementary Fig. 2D), while sporozoites expressing tdTomato under the *ef1α* promoter were only faintly fluorescent (Fig. 3B).

**Figure 4:**
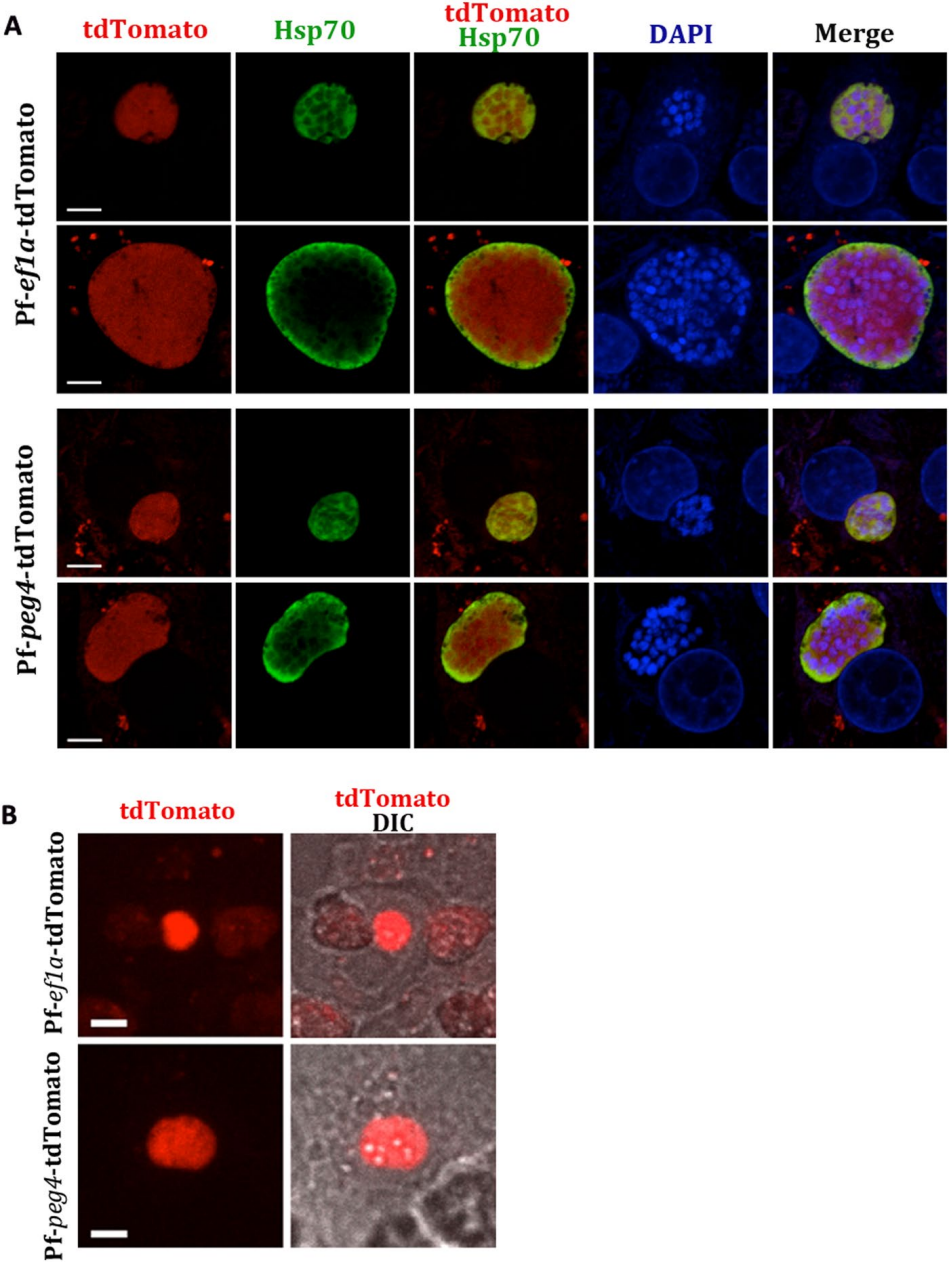
Immunofluorescene assays of late liver stage *P*. *falciparum*-tdTomato lines. (**A**) Primary human hepatocytes were infected with Pf-*peg4*-tdTomato and Pf-*ef1α*-tdTomato sporozoites. At 96 hours post infection, cells were fixed and stained for PfHsp70 (green) to visualize the parasite cytoplasm together with the endogenous tdTomato fluorescence (red). DNA was stained with DAPI (blue). Scale bars 5 μm. (**B**) Live confocal microscopy of Pf-*peg4*-tdTomato and Pf-*ef1α*-tdTomato EEFs at 96 hours post infection of primary human hepatocytes. Fluorescent images and overlays with the DIC images are shown. Scale bars 15 μm.

We also investigated the fluorescence of the transgenic tdTomato lines in the liver stage of the parasite by performing *in vitro* infection assays using sporozoites and primary human hepatocytes. Immunofluorescence assays of liver stage parasites co-stained with an mAb to heat shock protein 70 (hsp 70) and DAPI to visualize the DNA, showed that both the Pf-*ef1α*-tdTomato and Pf-*peg4*-tdTomato lines were brightly fluorescent in this stage (Fig. 4A). Furthermore, live imaging confirmed that liver stage parasites were brightly fluorescent and could be clearly seen without immunostaining (Fig. 4B). These data indicate that both lines could be used to study and isolate *P*. *falciparum* liver stage parasites.

**Figure 5:**
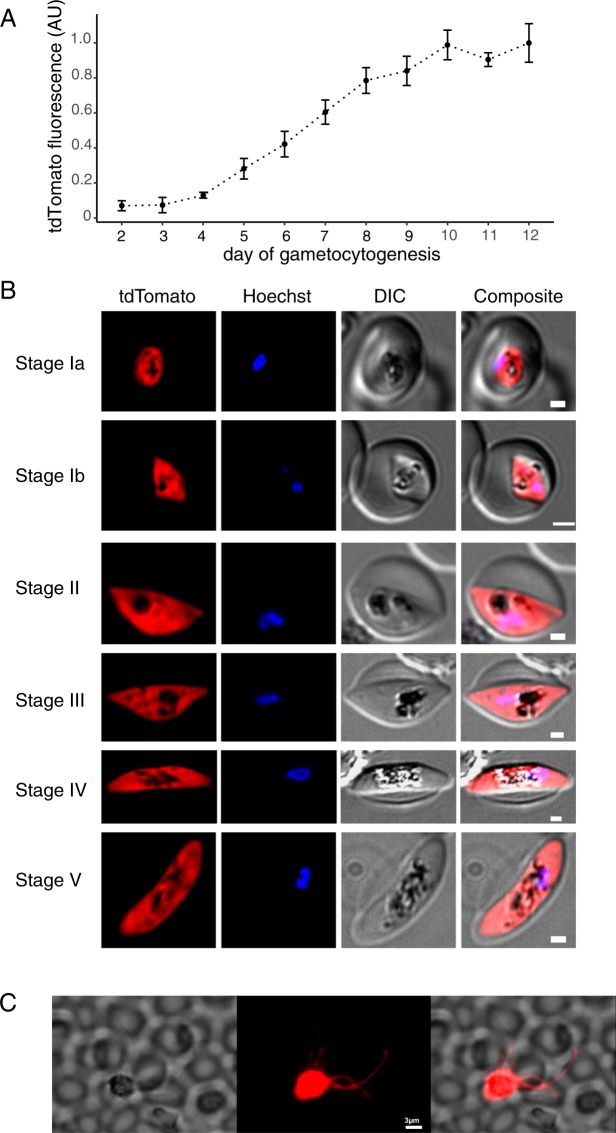
tdTomato expression increases throughout Pf-*peg4*-tdTomato gametocyte development. (**A**) Mean fluorescence of >1000 gametocytes was measured on days 2-12 by flow cytometry. Values are normalized to the maximum mean tdTomato signal on day 12. Mean +/− standard deviation from two independent experiments is shown. (**B**) Representative phase and fluorescence images of Pf-PEG4-tdTomato stage I-V gametocytes with the nuclear stain Hoechst 33342. Scale bar = 1 micron. (**C**) Exflagellating Pf-*peg4*-tdTomato microgametes. Scale bar = 3 microns.

As mentioned above, PEG4 is likely the *P*. *falciparum* ortholog of the rodent malaria parasite protein UIS4^8^, whose expression is highly upregulated in sporozoites and early liver stages^15,16^. In contrast to the rodent parasites, *P*. *falciparum* PEG4 is also expressed in gametocytes^6^. We therefore monitored the fluorescence of Pf-*peg4*-tdTomato gametocytes over the entire course of their development. We used flow cytometry to obtain fluorescence data from large numbers of gametocytes. Gametocytes could be clearly distinguished based on red fluorescence alone from stage III onward (day 5 of maturation). The fluorescence continued to increase as the gametocytes matured, reaching peak expression in mature stage V gametocytes (day 12) (Fig. 5A and Supplementary Fig. 3B). Images of gametocytes over the course of their development illustrate the expression of the tdTomato transgene in the different gametocyte stages of the Pf-*peg4*-tdTomato line (Fig. 5B), beginning as early as Stage I gametocytes (day 2 of maturation). Furthermore, exflagellation of male gametocytes could also be visualized (Fig. 5C). Thus this parasite line will be useful to laboratories studying the sexual stages of *P*. *falciparum* and could expedite the screening of drugs targeting gametocyte development.

In conclusion, we have generated three fluorescent *P*. *falciparum* lines with chromosomally-integrated transgenes that are infective for the mosquito host. While the Pf-ef1a-tdTomato line produces a constitutive fluorescent signal throughout the cell cycle, the Pf-*peg4*-tdTomato line produces highly fluorescent gametocytes and sporozoites that are sufficiently bright to be used in flow cytometry and intravital imaging experiments. The Pf-peg4-tdTomato sporozoites could be used in combination with humanized mice^17^ to gain information on the behavior of human malaria sporozoites *in vivo*. To date, these types of studies have only been performed with rodent malaria parasites^9,18^. Furthermore, the Pf-peg4-tdTomato sporozoites and liver stages of both tdTomato lines are sufficiently bright to test inhibitors of sporozoite motility and liver stage development using high content imaging to score the results. The use of the *peg4* promoter to drive tdTomato in gametocytes combines the best aspects of previous generations of fluorescent gametocyte reporter lines^7,19,20^, namely chromosomal integration, a strong promoter driving a bright red-fluorescent protein, and infectivity for the mosquito host. Additionally, the ability of flow cytometry to integrate stage-specific fluorescence reporters with measurements of DNA, RNA and hemozoin content, as well as mitochondrial membrane potential, will allow for multi-level cytological profiling of mixed stage cultures and more complex analyses of the impact of inhibitors on gametocyte maturation^21^. Overall, these lines will enable more rapid screening of both chemical inhibitors and potentially inhibitory antibodies on transmission stages of *P*. *falciparum* and aid in flow sorting transmission stages for downstream analyses such as transcriptomic and proteomic studies.

## Methods

### Asexual parasite culture and maintenance

Asexual blood stage parasites were propagated in 2% human erythrocytes in RPMI-1640 malaria culture medium containing 2 mM L-glutamine, 50 mg/L hypoxanthine, 25 mM HEPES, 0.225% NaHCO_3_, 10 mg/L gentamycin and 0.5% (w/v) Albumax II (Invitrogen). Parasites were maintained at 37 °C under an atmosphere of 5% O_2_, 5% CO_2_ and 90% N_2._

### Generation of the NF54^Pfs47attB^*Plasmodium falciparum* line

Zinc-finger nucleases (ZFN) directed against the *P*. *falciparum 6-cysteine protein P47* (PlasmoDB ID PF3D7_1346800, also known as *Pfs47*) were purchased from Sigma Life Sciences. To introduce the *attB* site (from *Mycobacterium smegmatis*) into the *pfs47* locus in *P*. *falciparum*, we generated a transfection plasmid with *pfs47* specific ZFNs, a selectable human *dhfr* marker and a donor fragment consisting of two *pfs47* homologous sequences flanking the *attB* site. The left and right ZFNs were 2A-linked and placed under the regulatory control of the 5′ *calmodulin* (PF3D7_1434200) promoter and the 3′ *hsp86* (PF3D7_0708500) terminator^22^. The two homologous regions flanking the ZFN cut site were first amplified separately using the primer pairs p1 and p2 (0.68 kb) and p3 + p4 (0.87 kb). Primer p2 and p3 also contained the *attB* site and the two fragments were then linked by “splicing by overlap extension” PCR. The donor sequence was added to the ZFN-containing plasmid using the restriction enzymes AatII and BstAPI, thus yielding the final pZFN^pfs47^-attB-h*dhfr* transfection plasmid. Asexual NF54 blood-stage parasites were electroporated with purified circular plasmid pZFN^*pfs47*^-attB-h*dhfr* as described^23^. One day after electroporation, parasites were exposed to 2.5 nM WR99210 for 6 days to select for transformed parasites. Integration of the *attB* site was assessed by PCR analysis of bulk cultures, and cultures showing evidence of editing were selected for 96-well cloning by limiting dilution. Clones were identified by staining with 2× SYBR Green I and 165 nM MitoTracker Deep Red (Invitrogen) and assaying for growth by flow cytometry on an Accuri C6 cytometer^24^. To test for *attB* integration, *P*. *falciparum* genomic DNA was extracted and purified using DNeasy Blood kits (Qiagen). *Pfs47* sequences were examined by PCR-amplifying the genomic locus with primers p5 + p6 flanking the *pfs47* plasmid donor sequence. These products were amplified from bulk cultures or parasite clones. Sequencing was performed with the internal p7 and p8 primers. Four clones were expanded to test their ability to infect mosquitoes and of these, NF54^Pfs47attB^ C10 gave the most robust mosquito infections and was therefore used for production of the fluorescent lines. Primer sequences in Table S1.

### Generation of fluorescent *P*. *falciparum* lines

Plasmid construction was performed using standard molecular cloning techniques. The *Pf-ef1α* used was the same 806 bp sequence described and used by Talman *et al*.^2^, and was amplified by genomic DNA using primers p9 and p10. The 2227 bp *Pf-peg4* upstream sequenced used was identical to that described by Buchholz *et al*.^19^, and was amplified from genomic DNA using primers p11 and p12. The *Pf-csp* sequence used consisted of a 1266 bp sequence from upstream of the *csp* coding sequence (PF3D7_0304600). The final plasmids were denoted pBattP-eEF1apr_tdTomato, pBattP_PEG4pr_tdTomato, and pLN_CSpr-GFP. Transfections were performed with 50 μg of the fluorescent protein plasmid along with 50 μg of the pINT integrase plasmid (genbank plasmid DQ813654.3)^14^ using the modified method described by Spalding *et al*.^25^. Transfections were selected with 2.5 μg/mL Blasticidin S until stable parasites were obtained. Transfected lines were cloned by limiting dilution as described above. Chromosomal integration of the plasmids was verified by PCR using primers p13 and p15, and compared to PCR of the unmodified pfs47attB locus with primers p13 and p14. Primer sequences in Table S1.

### Gametocyte cultures and mosquito feeding

Gametocytes cultures were maintained as described above except with the following modifications: Culture medium contained 10% *v/v* human serum in the place of Albumax II, and parasites were propagated at 4% hematocrit. Instead of a gas incubator, cultures were maintained at 37 °C in a candle jar made from glass desiccators. Gametocyte cultures were initiated at 0.5% asynchronous asexual parasitemia from a low passage stock and maintained up to day 18 with daily media changes but without any addition of fresh erythrocytes. Day 15 to 18 cultures, containing largely mature gametocytes, were used for mosquito feeds: Cultures were centrifuged at 108 × *g* for 4 min and the parasite pellet was resuspended to final gametocytemia of 0.3% in a mixture of human O^+^ RBCs supplemented with 50% *v/v* human serum. Gametocytes were fed to *Anopheles stephensi* mosquitoes (Liston strain) that had been starved overnight, using glass membrane feeders. Unfed mosquitoes were removed after feeding. Fed mosquitoes were then maintained on a 10% sugar solution at 25 °C and 80% humidity with a 14:10 hr light:dark cycle including a 2 hr dawn/dusk transition. according to standard rearing methods. Human erythrocytes used to set up the cultures were  collected weekly from healthy donors under the Johns Hopkins University Institutional Review Board approved protocol number NA_00019050. All experiments were performed in accordance with institutional guidelines and regulations and informed consent was obtained from all volunteers.

### Oocyst and salivary gland sporozoite quantification

*An*. *stephensi* mosquitoes infected with Pf-*ef1α*-tdTomato, Pf-*peg4*-tdTomato or Pf-*csp*-GFP parasites were maintained at 25 °C, 80% humidity and on days 10 and 14 after the infective-blood meal, mosquitoes were dissected and midguts or salivary glands were harvested for sporozoite counts. On day 10 midguts were observed and photographed for oocyst counts by fluorescence and phase microscopy using an upright Nikon E600 microscope with a PlanApo 10× objective. On day 14, salivary glands from 30–50 mosquitoes were pooled, and released sporozoites were counted using a haemocytometer. For direct visualization of Pf-*ef1α*-tdTomato or Pf-*peg4*-tdTomato sporozoites *in situ*, infected mosquitoes were immobilized at 4 °C and transferred to a petri dish pre-chilled on ice. Mosquitoes were observed under an Olympus SZX7 fluorescence stereo microscope for detection of red fluorescence from transgenic sporozoites that had invaded the salivary glands inside the mosquito thoracic cavity. Images of the mosquitoes were obtained using the Q-Capture Pro 7 software. Uninfected mosquitoes were used as negative control.

### Immunofluorescence assays (IFAs)

#### Sporozoites

Pf-*ef1α*-tdTomato, Pf-*peg4*-tdTomato or Pf-*csp*-GFP sporozoites were isolated from infected mosquitoes 16 days post-infection. Sporozoites were centrifuged onto a glass coverslip at 300 × g for 4 min to increase attachment to the coverslip. Sporozoites were fixed with 4% paraformaldehyde for 10 min, washed with PBS and then incubated with Alexa Fluor 488 or Alexa Fluor 594 labeled mAb 2A10, specific for the *P*. *falciparum* circumsporozoite protein^26^ diluted in PBS to a concentration of 1 μg/ml for 1 hour at room temperature. After three washes with PBS, sporozoites were mounted with ProLong Gold Antifade with DAPI (Thermofisher) and visualized on a Zeiss Axio Imager M2 fluorescence microscope for detection of tdTomato red fluorescence, PfCSP green fluorescence and DAPI. Sporozoite images were obtained and analyzed using the Volocity software.

#### Exoerythrocytic stages

Cryopreserved human primary hepatocytes, hepatocyte thawing, plating and maintenance medium were obtained from Triangle Research Labs (TRL, North Carolina). Growth and infection of primary hepatocytes was performed as previously outlined in^27^. Briefly, primary hepatocytes (donor 4051) were thawed and 300,000 hepatocytes were plated on collagen-coated coverslips in a 24 well plate. 48 hrs later, 3-5 × 10^5^ Pf*-ef1α*-tdTomato or Pf*-peg4*-tdTomato sporozoites were added per well in Iscove’s Modified Dulbecco’s Medium (IMDM) with 2.5% FCS supplemented with penicillin, streptomycin and L-glutamine (complete growth medium), centrifuged at 380 × *g* for 5 min without break and then incubated at 37 °C for 4–6 hours. After incubation, cells were washed with PBS and maintained in complete growth medium supplemented with Fungizone (1.25 μg/ml, Gibco, Grand Island, NY). For IFAs, infected cells were fixed in 4% paraformaldehyde 96 hours post infection, washed in 1x PBS and permeabilized for 10 min at RT in 0.1% TritonX-100/PBS and incubated for 15 min in FX Signal Enhancer (Thermofisher #136933). Samples were blocked for 1 hour in 10% BSA/10% goat serum/0.1% TritonX100 and stained mAb 4C9 specific for Pf Hsp70^28^, followed by anti-mouse IgG-Alexa Fluor 488. Images were acquired using a LSM700 laser scanning confocal microscope (Zeiss AxioObserver, Carl Zeiss AG, Oberkochen, Germany) with a 63x PlanApo oil objective, and images were acquired using ZEN software (Carl Zeiss AG, Oberkochen, Germany). For live imaging, cells were directly imaged using an inverted Zeiss Axio Observer Z1 microscope with a Yokogawa CSU22 spinning disk using an EMCCD camera and 3i slidebook 5.0 software.

### Gametocyte assays

Pf-*peg4*-tdTomato gametocytes were induced synchronously according to previous methods^29^ and asexual stages were eliminated by treatment with 50 mM GlcNAc (Alfa Aesar, Haverhill, MA) and 20 U/ml heparin (Sigma) for 3 days following re-invasion. On days 2-12 of gametocytogenesis, cultures were stained with 16 μM Hoechst33342 (Invitrogen) and 50 nM DilC1(5) (Invitrogen) for 30 min at 37 °C. Mean tdTomato fluorescence was measured using a Cytek DxP12 flow cytometer for >1000 viable gametocytes, gated as infected (Hoechst 33342-high), hemozoin-containing (depolarized side scatter-high) RBCs with positive membrane potential (DilC1(5)-high). This gating strategy is illustrated in Supplementary Fig. 3A. Fluorescence images of stained gametocytes were acquired at 1000x magnification using a Leica DM-6000B. tdTomato and Hoechst 33342 Z-stacks were deconvoluted using the non-linear least square algorithm of the Deconvolution Lab2 plug-in of ImageJ and representative slices are shown along with DIC brightfield images. Image of tdTomato-positive microgametes was acquired at 600x magnification after treating cultures of Stage V (day 12) gametocytes with 20 µM Xanthurenic Acid (Sigma) at room temperature.

## Supplementary information

Supplementary Information
