# Supplementary material for: Generation of Transmission-Competent Human Malaria Parasites with Chromosomally-Integrated Fluorescent Reporters
Source: Sci Rep. 2019 Sep 11;9:13131. doi: 10.1038/s41598-019-49348-x (PMC6739413; doi:10.1038/s41598-019-49348-x)
Supplement: Supplementary file 1 — Supplementary Information [file 41598_2019_49348_MOESM1_ESM.pdf]

Supplemental Information

Generation of Transmission-Competent Human Malaria Parasites with Chromosomally-Integrated Fluorescent Reporters

Kyle McLean, Judith Straimer, Christine S. Hopp, Joel Vega-Rodriguez, Jennifer L. Small-Saunders, Sachie Kanatani, Abhai Tripathi, Godfree Mlambo, Peter C. Dumoulin, Chantal T. Harris, Xinran Tong, Melanie J. Shears, Johan Ankarklev, Björn F.C. Kafsack, David A. Fidock, and Photini Sinnis

Contents

Supplemental Figure 1. Construction of the Pf-*ef1α*-tdTomato line

Supplemental Figure 2. Phenotypic characterization of the uncloned Pf-*csp*-GFP line

Supplemental Figure 3. Schematic of gametocyte gating strategy and distribution of tdTomato fluorescence over gametocyte maturation

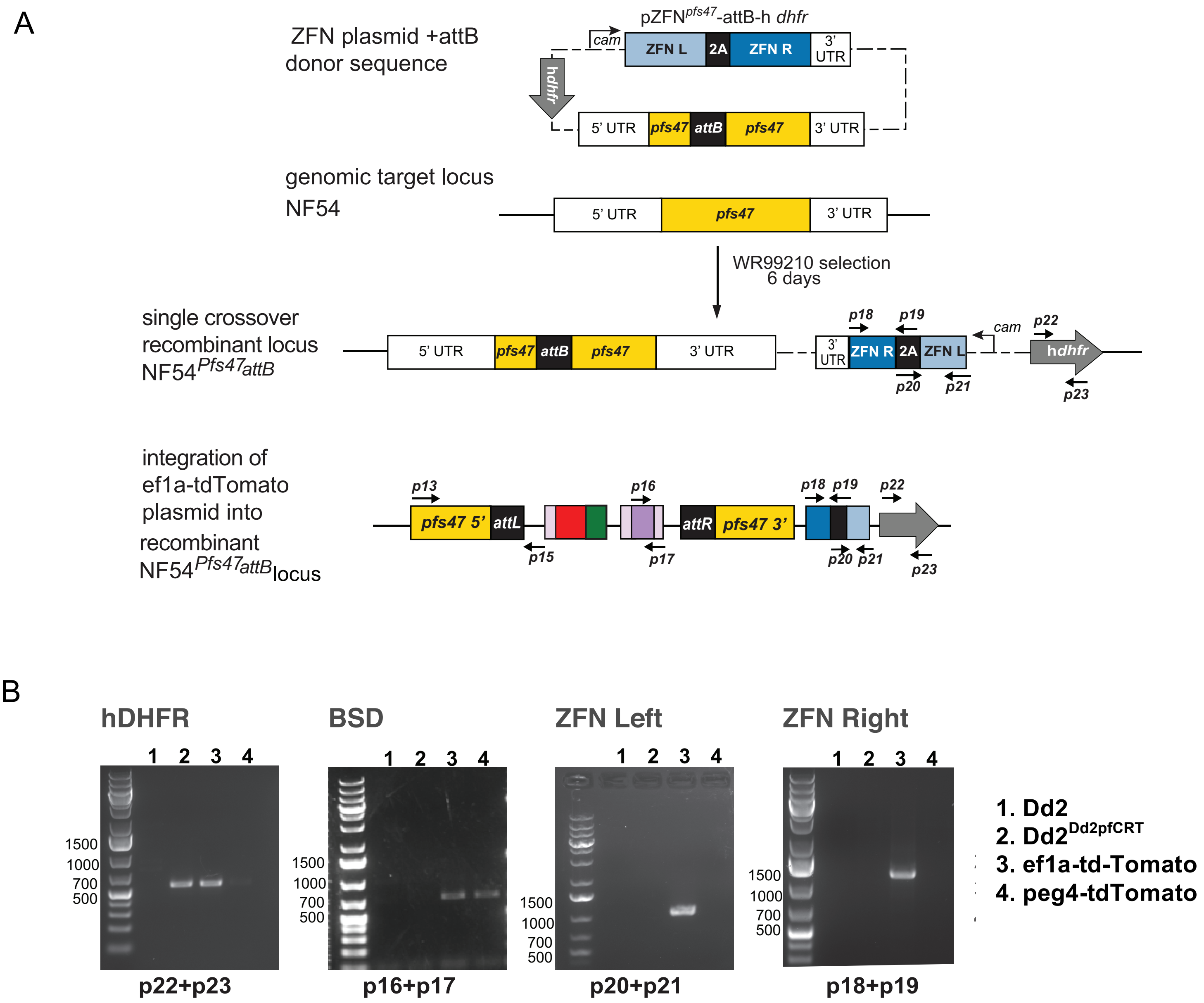

Supplementary Figure 1. Construction of the Pf-*ef1α*-tdTomato line. (A) Schematic of the single crossover event that likely occurred in the ef1α-tdTomato line. The eEF1α promoter-driven tdTomato plasmid shown in Figure 1 was transfected into NF54 Pfs47attB parasites that had undergone single-site crossover with the original ZFN plasmid harboring the attB sequence and the hDHFR selection cassette. Integration of the eEF1α promoter-driven tdTomato plasmid occurred upon attB×attP recombination, resulting in a parasite that harbors both the hDHFR and BSD cassettes. This is in contrast with the Pf-*peg4*-tdTomato lines, for which the attB sequence was inserted via ZFN-mediated homology-directed recombination, as opposed to single-site crossover. Pf-*peg4*-tdTomato parasites harbor only the attB cassette and not hDHFR. (B) Genomic DNA was isolated from the indicated parasites and PCRs were designed to amplify the DNA sequences specified above. Primer locations are shown in panel A. Gels were loaded as follows: lane 1, Dd2; lane 2, Dd2^{Dd2pfCRT}; lane 3, Pf-*ef1α*-tdTomato Clone 3D06; lane 4, Pf-*peg4*-tdTomato Clone A. Dd2^{Dd2pfCRT} parasites were generated via ZFN-mediated gene editing of the *pfcrt* locus. This line harbors the hDHFR but not the BSD cassette, and lacks residual ZFNs. Primer sequences in Table S1.

A

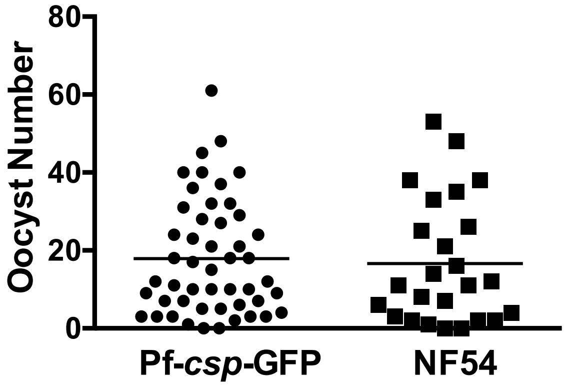

B

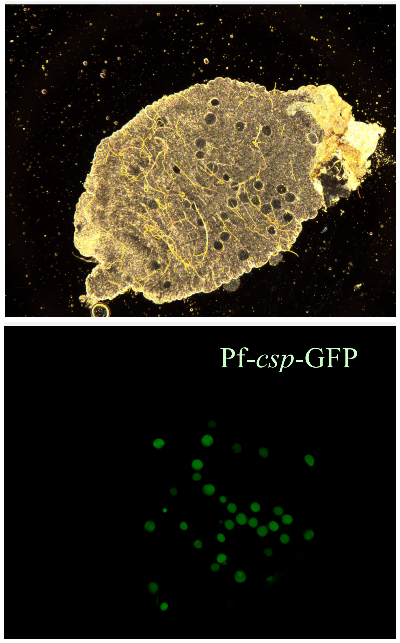

C

Sporozoites per salivary gland	
Pf-*csp*-GFP cycle 1	38,500
Pf-*csp*-GFP cycle 2	26,470
Pf-*csp*-GFP cycle 3	35,000

D

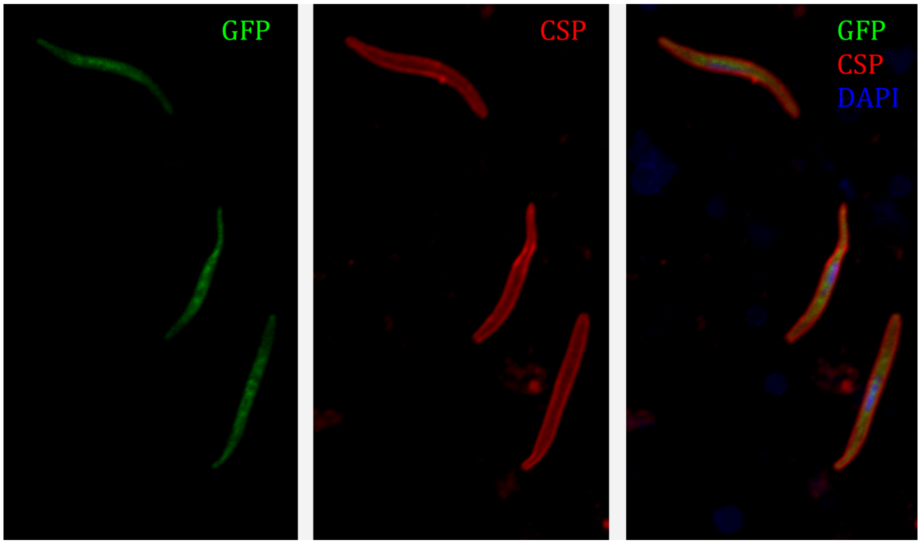

Supplementary Figure 2. Phenotypic characterization of the uncloned Pf-*csp*-GFP line. (A) Oocyst and salivary gland sporozoite numbers. Oocysts were counted on day 10 post-infectious blood meal and numbers are shown from 3 independent mosquito infections with the Pf-*csp*-GFP line (total n=49 midguts). Two of these cycles were performed in parallel with the tdTomato lines shown in Figure 2B and one cycle was performed in parallel with control NF54 parasites shown above (n=25 midguts). (B) Phase and fluorescence image of an infected midgut. Though the line was not cloned, the majority of oocysts were fluorescent. (C) Salivary gland sporozoites were counted on day 14 post-infectious blood meal; numbers shown are the mean of salivary gland loads from 20 mosquitoes from each of the above mosquito cycles. (D) Immunofluorescence image of salivary gland sporozoites fixed and stained for PfCSP (red) to visualize the plasma membrane of the sporozoite. The endogenous GFP fluorescence appeared to be primarily cytoplasmic (green). DNA was stained with DAPI (blue).

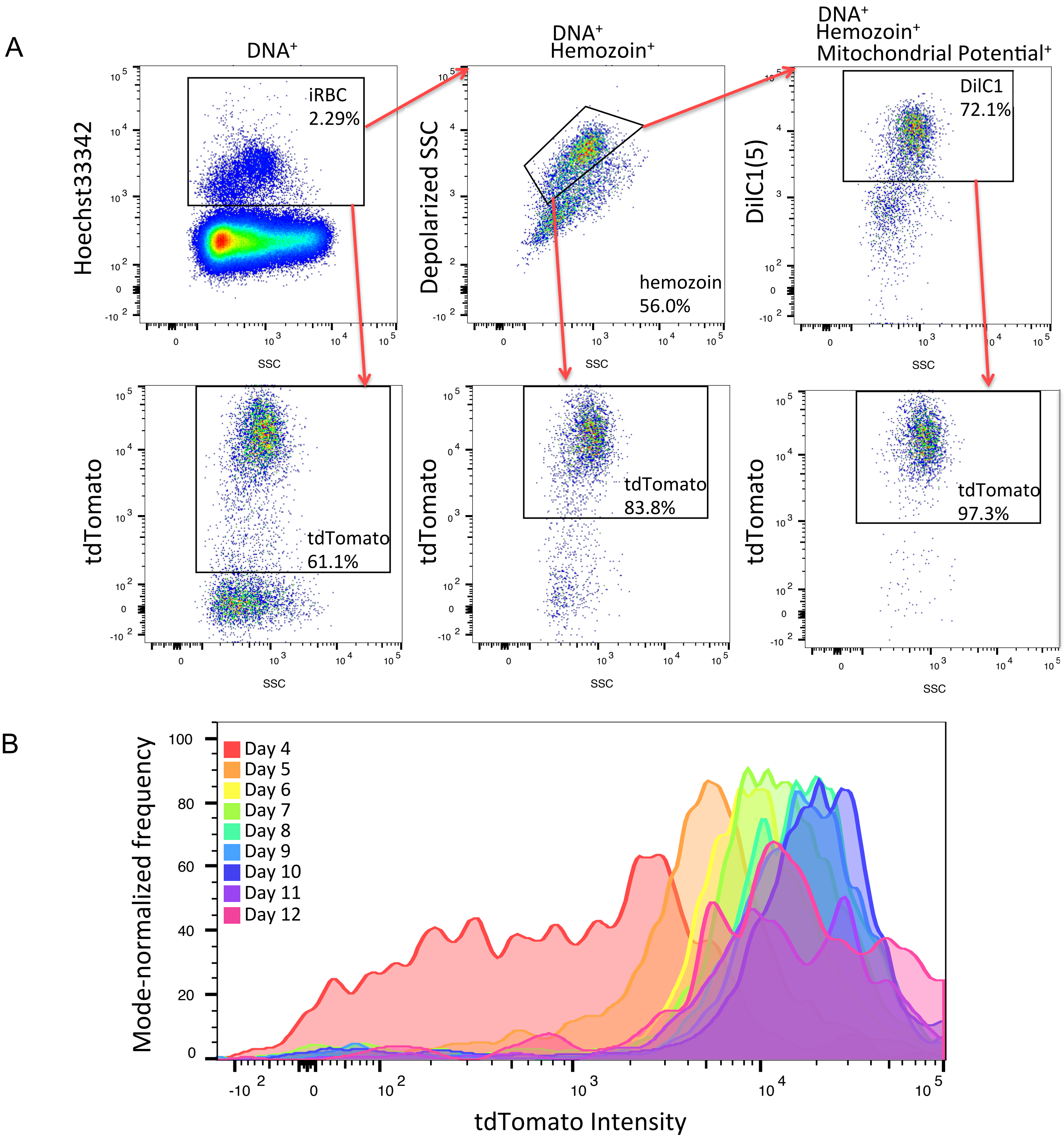

Supplementary Figure 3. Schematic of gametocyte gating strategy and distribution of tdTomato fluorescence over gametocyte maturation. (A) Fluorescence in live gametocytes was measured by gating for DNA, hemozoin, and mitochondrial membrane potential. The example shown is for day 8 gametocytes. (B) Distribution of tdTomato fluorescence in gametocytes on days 4-12 of development. Frequency was normalized to the mode on each day.

Supplementary Table 1. List of primers used in this study.

Primer	Sequence (5'-3')	Lab number
p1	gtgacgtcGGAGTTCTACATTAATGCAACTGTCC	p3739
p2	GATGATCCTGACGACGGAGACCGCCGTCGTCGACAAGCCGagatctaagcttctaATCACATACGTATTGTGTTGAGC	p3538
p3	GTCGACGACGGCGGTCTCCGTCGTCAGGATCATCGCGGaagcttGGATGTACTATAAACAACCCTACG	p3539
p4	ATATGGACATTTAATGCCTGC	p3738
p5	CACGCACTTTTTTTATTACAATGATCGC	p4714
p6	GGTAAGGCTAACATATACATGCCTTCC	p4715
p7	GTGTATGGGAAGAATGATCAGC	p3798
p8	CTAAATGATATGCGCTGGAATC	p3799
p9	TTGAATTCTTTAACGGTTCACCCCTCTTAACC	KM0113
p10	TTCTCGAGGTTTAGTATATTAATATATATGTATA	KM0114
p11	TTGAATTCGCATGAACGTTTTGTAAAC	KM0111
p12	TTCTCGAGGTCGGAAATCGGATAAGAAG	KM0112
p13	GTGTATGGGAAGAATGATCAGC	KM0740
p14	CTAAATGATATGCGCTGGAATC	KM0741
p15	GTCGGGGCTGGCTTAACTAT	KM0742
p16	GGTGATAAATGCATGCCAAGCCTTTG	p7845
p17	CAGACAGTAAAAAAAATCGCTATCCCATAAATTACAAAACATG	p7848
p18	GAGTTATCAGAAGTTGATCTCGCCGTTG	p7826
p19	TTCTAACCTGCGGTGACGTG	p7828
p20	CACGTCACCGCAGGTTAGAA	p7827
p21	CCATGACGGTGATTATAAAGATCATGAC	p7829
p22	ATGGTTGGTTCGCTAAACTGCATCGT	p7279
p23	gaattcTTAATCATTCTTCTCATATACTTCAAATTTG	p3316
